# Genetic Predisposition to Mosaic Chromosomal Loss Is Associated With Functional Outcome After Ischemic Stroke

**DOI:** 10.1212/NXG.0000000000000634

**Published:** 2021-11-12

**Authors:** Malin Johansson, Annie Pedersen, John W. Cole, Cecilia Lagging, Arne Lindgren, Jane M. Maguire, Natalia S. Rost, Martin Söderholm, Bradford B. Worrall, Tara M. Stanne, Christina Jern

**Affiliations:** From the Institute of Biomedicine (M.J., A.P., C.L., T.M.S., C.J.), Sahlgrenska Academy at the University of Gothenburg; Department of Clinical Genetics and Genomics (A.P., C.L., C.J.), Sahlgrenska University Hospital, Gothenburg, Sweden; Department of Neurology (J.W.C.), Baltimore VA Medical Center and University of Maryland School of Medicine, Baltimore, MD; Department of Clinical Sciences Lund (A.L., M.S.), Neurology, Lund University; Department of Neurology (A.L., M.S.), Skåne University Hospital, Lund and Malmö, Sweden; Faculty of Health (J.M.M.), University of Technology Sydney, Australia; Hunter Medical Research Centre (J.M.M.), Newcastle, Australia; J. Philip Kistler Stroke Research Center (N.S.R.), Department of Neurology, Massachusetts General Hospital, Harvard Medical School, Boston; and Departments of Neurology and Health Evaluation Sciences (B.B.W.), University of Virginia, Charlottesville, VA.

## Abstract

**Background and Objectives:**

To test the hypothesis that a predisposition to acquired genetic alterations is associated with ischemic stroke outcome by investigating the association between a polygenic risk score (PRS) for mosaic loss of chromosome Y (mLOY) and outcome in a large international data set.

**Methods:**

We used data from the genome-wide association study performed within the Genetics of Ischemic Stroke Functional Outcome network, which included 6,165 patients (3,497 men and 2,668 women) with acute ischemic stroke of mainly European ancestry. We assessed a weighted PRS for mLOY and examined possible associations with the modified Rankin Scale (mRS) score 3 months poststroke in logistic regression models. We investigated the whole study sample as well as men and women separately.

**Results:**

Increasing PRS for mLOY was associated with poor functional outcome (mRS score >2) with an odds ratio (OR) of 1.11 (95% confidence interval [CI] 1.03–1.19) per 1 SD increase in the PRS after adjustment for age, sex, ancestry, stroke severity (NIH Stroke Scale), smoking, and diabetes mellitus. In sex-stratified analyses, we found a statistically significant association in women (adjusted OR 1.20, 95% CI 1.08–1.33). In men, the association was in the same direction (adjusted OR 1.04, 95% CI 0.95–1.14), and we observed no significant genotype-sex interaction.

**Discussion:**

In this exploratory study, we found associations between genetic variants predisposing to mLOY and stroke outcome. The significant association in women suggests underlying mechanisms related to genomic instability that operate in both sexes. These findings need replication and mechanistic exploration.

Outcomes after ischemic stroke exhibit a large interindividual variation, only partially explained by clinical factors. There is a need to better understand the molecular mechanisms underlying this variation.

It is well known that chronological age is a strong predictor of poor stroke outcomes. In recent years, it has been increasingly recognized that human aging is accompanied by gradual accumulation of acquired (i.e., somatic) mutations, including chromosomal aberrations, in cells from different tissues. In blood, clonally expanded cells with acquired genetic alterations are denoted clonal hematopoiesis (CH). There are several varieties of CH including single nucleotide variants and mosaic chromosomal alterations (mCAs, i.e., autosomal or sex chromosome), and the most common is mosaic loss of chromosome Y (mLOY) in men.^1^ Of note, there is support for an underlying genetic overlap between different subtypes of CH.^1^

Acquired genetic alterations have been shown to play a role in aging and age-related diseases.^2^ CH is associated with an increased risk of overall mortality as well as with common diseases of aging such as cancer and cardiovascular disease.^1,3^ For mLOY specifically, an increased proportion of leukocytes with mLOY has been associated with shorter lifespan, higher risk of nonhematologic cancers, Alzheimer disease, and cardiovascular disease including stroke and has been proposed as a marker of biological aging.^4-8^

Recently, a genome-wide association study (GWAS) identified over 150 autosomal loci associated with mLOY in leukocytes.^9^ Many of the implicated genes are involved in various aspects of cell cycle regulation and the DNA damage response, such as mitotic structure formation, replication and stability of DNA, cell arrest, and apoptosis.^9^ Notably, a polygenic risk score (PRS) that included these variants was associated with breast cancer and age at menopause.^9^ This implies that the genetic predisposition to mLOY is a biomarker of a broader genomic instability that is of relevance for both sexes. In line with this, a recent study showed that this PRS for mLOY was also associated with X chromosome and autosomal mCA in a large cohort of participants from 5 biobanks including the UK biobank.^10^ Given this background, we hypothesized that the genetic loci associated with mLOY, through a predisposition to an increased general genomic instability, are of plausible relevance for processes that affect brain recovery after stroke. We therefore investigated this PRS for mLOY for association with functional outcome after ischemic stroke.

## Methods

### Study Population

This study comprised the ischemic stroke cases included in the first GWAS performed within the Genetics of Ischemic Stroke Functional Outcome (GISCOME) network, which has been described previously.^11,12^ In brief, 6,165 cases with acute ischemic stroke of mainly European ancestry from 12 different study locations were included. Stroke severity at index stroke was assessed with the NIH Stroke Scale and 3-month functional outcome with the modified Rankin Scale (mRS). For some of the Swedish study participants, outcome data were obtained from the national register Riksstroke which does not discriminate between the mRS scores 0, 1, and 2. To be able to include all study participants in this analysis, we therefore dichotomized the mRS score at 0–2 vs 3–6. Another reason for choosing this cutoff is that it reflects being dependent or independent in activities of daily living and is thus a clinically important difference in functional outcome. Genotyping was performed with single nucleotide polymorphism arrays with subsequent imputation to the 1000 Genomes Phase 3 reference panel, as previously described.^11^ Each center obtained ethical approval and participant consent individually. Additional details can be found in the Supplemental Methods.

### Polygenic Risk Score

We used individual genotypic data from the GISCOME GWAS to calculate a weighted PRS for each study participant. For this, we used the PRS for mLOY, which includes 156 independent genetic variants and this PRS has been described in detail elsewhere.^9^ Of the reported variants, 115 were available (directly genotyped or imputed) in our data set. For missing variants, we used LDlink^13^ to search for proxies (r^2^ > 0.8), which resulted in inclusion of 12 additional variants. Thus, we included 127 variants in total (eMethods, links.lww.com/NXG/A486; eTable 1, links.lww.com/NXG/A485). To calculate the PRS, we multiplied natural log transformed odds ratios (ORs) for previous association to mLOY (eMethods; eTable 1) for each of the 127 variants by the number of risk alleles. The sum of the products resulted in an individual PRS.

### Statistical Analyses

Baseline characteristics were compared using the χ^2^ test for proportions and the Mann-Whitney *U* test for numeric variables. We assessed associations between the PRS and stroke outcome by 4 logistic regression models, all adjusted for ancestry (the first 5 principal components). Models 2, 3, and 4 were additionally adjusted for age at index stroke and sex, models 3 and 4 also for stroke severity, and model 4 also for smoking and diabetes mellitus. The reason that model 4 included smoking and diabetes is that these are the vascular risk factors that have been reported to associate with mLOY.^5,14^ We performed prespecified sex-stratified analyses, and genotype-sex interactions were further assessed in the logistic regression models. We also performed sensitivity analyses restricted to survivors at 3 months (mRS scores 0–2 vs 3–5, i.e., excluding mRS score 6) as well as conditional regression analyses on a subgroup of cases with good and poor outcome matched for age (±1 year) and sex. To exclude the possibility of a few variants excessively driving any detected association, we performed sensitivity analyses, modifying the PRS by including different compositions of the mLOY-associated variants.

All statistical analyses were performed using R version 3.6.3. A 2-tailed *p* < 0.05 was considered statistically significant.

### Data Availability

The data sets generated and analyzed during the current study are available on reasonable request.

## Results

Baseline characteristics for the study sample are shown in the Table. As expected, individuals with poor functional outcome were older and had more severe strokes and a higher proportion of diabetes mellitus (24% in the poor vs 18% in the good outcome groups). On the contrary, the proportion of smokers was lower in the poor outcome group (18% vs 26%), which might be explained by a lower median age in smokers. Baseline characteristics according to PRS tertiles of the whole sample, the good vs poor outcome groups, and men vs women are shown in eTable 2 (links.lww.com/NXG/A485) (eMethods, links.lww.com/NXG/A486). There were no significant differences in baseline characteristics between tertiles 1 and 3, except that men in the lowest tertile were slightly older (*p* = 0.04) and had a lower proportion of diabetes mellitus (*p* = 0.01).

**Table:**
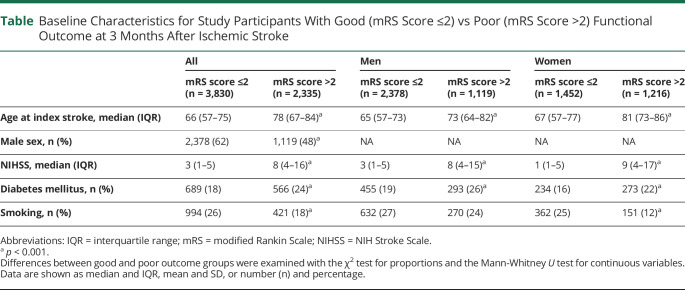
Baseline Characteristics for Study Participants With Good (mRS Score ≤2) vs Poor (mRS Score >2) Functional Outcome at 3 Months After Ischemic Stroke

Higher PRS was significantly associated with poor poststroke functional outcome with an OR of 1.06 (95% confidence interval [CI], 1.00–1.12, *p* = 0.020) per 1 SD (SD) increase in PRS after adjustment for ancestry and an OR of 1.07 (95% CI 1.01–1.13, *p* = 0.020) after adjustment for ancestry, age, and sex (Figure 1A). When including stroke severity in the model, the OR was 1.11 (95% CI 1.04–1.18, *p* = 0.003). Results were similar after additional adjustment for smoking and diabetes mellitus, OR 1.11 (95% CI 1.03–1.19, *p* = 0.004) (Figure 1A). We noted that all covariates were significantly associated with outcome in the models (*p* < 0.05, data available from Dryad; eTable 3, links.lww.com/NXG/A485). When restricting the analyses to survivors to rule out an association to mortality driving the findings, the observed associations were similar, e.g., OR 1.09 (95% CI 1.02–1.15, *p* = 0.006) after adjustment for ancestry, age, and sex. Sensitivity analyses including different compositions of the mLOY associated variants did not indicate that a few SNPs excessively influenced the observed associations in our sample (data not shown). To address a potential residual confounding by age, we additionally analyzed the data on a subgroup of 3,386 cases matched for age and sex using conditional logistic regression. These analyses yielded similar results as the original analyses in all models (OR 1.10 [95% CI 1.03–1.18], *p* = 0.01 after adjustment for ancestry, 1.17 [1.06–1.29], *p* < 0.01 after adjustment for ancestry and stroke severity, and 1.15 [1.04–1.28], *p* = 0.01, after adjustment for ancestry, stroke severity, smoking, and diabetes mellitus).

**Figure:**
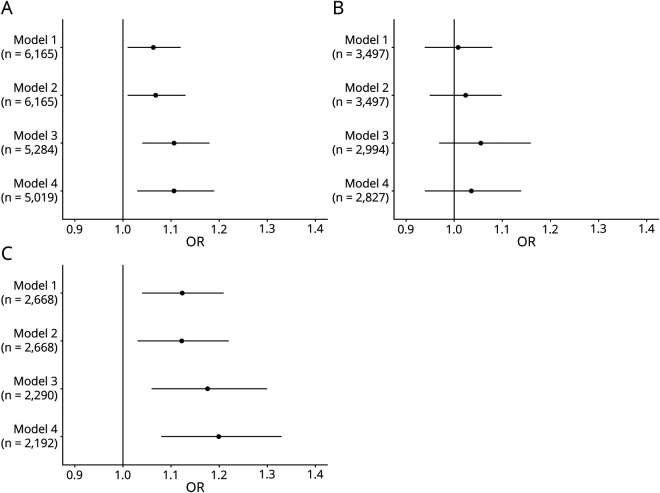
Forest Plots Showing Associations Between a PRS for mLOY in Leukocytes and Functional Outcome at 3 Months After Index Ischemic Stroke (A–C) Forest plots showing OR and 95% CIs for poor 3-month outcome (mRS score >2) per 1 SD increase in the PRS. (A) All ischemic stroke cases (N = 6,165), (B) men only (N = 3,497), and (C) women only (N = 2,668). Model 1, adjusted for ancestry (the first 5 PCs); model 2, adjusted for age at index stroke, sex, and ancestry (the first 5 PCs); model 3, adjusted for age at index stroke, sex, ancestry (the first 5 PCs), and stroke severity (NIHSS); model 4, adjusted for age at index stroke, sex, ancestry (the first 5 PCs), stroke severity (NIHSS), smoking, and diabetes mellitus. CI = confidence interval; MAF = minor allele frequency; mLOY = mosaic loss of chromosome Y; mRS = modified Rankin Scale; NIHSS = NIH Stroke Scale; OR = odds ratio; PC = principal component; PRS = polygenic risk score.

The male:female ratio in the study population was 1.3:1.0. Women were older (*p* < 0.01) and had more severe strokes (*p* < 0.01) compared with men, whereas the proportions of patients smoking and having diabetes mellitus, respectively, were higher in men (26% vs 19%, *p* < 0.01 and 21% vs 19%, *p* < 0.05, respectively). In the sex-stratified analyses, there was an association between increasing PRS and poor functional outcome in women (OR 1.12 (95% CI 1.03–1.22, *p* = 0.007) per 1 SD increase in the PRS) after adjustment for ancestry and age. The ORs increased slightly after additional adjustment for stroke severity (1.18, 95% CI, 1.06–1.30, *p* = 0.002) and smoking and diabetes mellitus (1.20, 95% CI, 1.08–1.33, *p* = 0.001) (Figure 1C). In men, the association between the PRS and functional outcome was in the same direction, but the associations were not statistically significant in any of the logistic regression models (Figure 1B). However, a genotype-sex interaction term was not statistically significant (*p* = 0.14).

## Discussion

In this exploratory analysis, we found that autosomal loci predisposing to loss of chromosome Y were associated with worse functional outcome after ischemic stroke. In sex-stratified analyses, this association was statistically significant in women. In men, the association was in the same direction, and we observed no significant genotype-sex interaction. A larger, well-powered study would permit better exploration of these potential sex-specific associations.

There is support that mLOY in leukocytes is a biomarker reflecting a broader predisposition to genomic instability that may occur in parallel in other tissues. Genetic determinants of mLOY have shown associations with X-chromosome loss in women as well as with mCA (both autosomal and X chromosome).^10,15^ Furthermore, several of the genetic variants that predispose men to mLOY (in particular those involved in the DNA damage response and telomere maintenance pathway) also predispose to other CH subtypes in both sexes (i.e., mCAs and CH of indeterminate potential [CHIP]).^1^ In line with these results, genetic determinants of mLOY have shown to be associated with diabetes mellitus and nonhematologic cancers in both men and women, as well as age at menopause in women.^9,14^ Given previous support for associations between several subtypes of CH and age-related diseases including cardiovascular disease, a mechanism through CH might explain the present observed association between the PRS for mLOY and functional outcome after ischemic stroke. Genomic instability leading to an increased number of somatic mutations in other tissues, such as the brain, is another mechanism that plausibly could influence stroke recovery. With regard to stroke outcomes, there is previous support that germline copy number variations influence functional outcome at 3 months poststroke.^16^ Further studies are needed to decipher whether these reflect shared or distinct underlying mechanisms.

Genomic instability resulting in CH may in turn predispose to immunologic alterations. Previous studies propose that CH resulting in blood cells lacking chromosome Y may lead to dysregulation of disease-protective immune functions and thus an increased risk for disease development.^4,14^ This view was supported by results from a study indicating impaired immunosurveillance as the mechanism underlying associations between mLOY in blood and increased risk of incident Alzheimer disease.^7^ A very recent study that investigated DNA and RNA from leukocytes in sorted and single cells in vivo and in vitro found that loss of chromosome Y (LOY) was associated with dysregulation of ∼ 500 autosomal genes, for example, those involved in immune functions but also with roles in other biological processes.^17^ Similarly, immunologic mechanisms could affect stroke outcome by affecting processes implicated in recovery or by affecting the general condition of the brain. The relation between mLOY and immune function was recently further supported by a large population-based study showing that mLOY in circulating blood cells was associated with changes in leukocyte, erythrocyte, and thrombocyte counts. Of interest, the same study found similar associations between the PRS for LOY and blood cell counts in both men and women.^18^

Many of the loci in the investigated PRS are linked to cell cycle regulation, proliferation, responses to DNA damage, and/or apoptosis.^9^ Genetic variants influencing these processes could be of relevance also for processes in the recovering brain after stroke,^19,20^ which could contribute to the observed association to stroke outcome. However, we did not detect a single or any combination of a few SNPs that excessively influenced the observed associations in our sample.

Finally, mLOY has been suggested to be a biomarker of biological aging,^7^ and a recent DNA methylation study indicated that biological aging is more strongly associated with stroke outcomes than chronological age.^21^ In this context, it is of note that the PRS investigated here includes variants in some telomere-related loci. However, these loci did not excessively influence the observed association. Furthermore, inclusion of chronological age in the regression models only marginally affected the observed associations between the PRS and stroke outcome, and chronological age was independently associated with stroke outcome in all investigated models. Thus, we found no support for this hypothesis in our results.

The GISCOME study represents the largest data set with both genetic and ischemic stroke outcome data available to date. However, it still constitutes a limited sample size that does not allow any clear conclusions on putative sex-specific associations. Another limitation is the lack of information on premorbid mRS, which would have provided a possibility to calculate the change in functional ability. We also lacked data on some important factors that influence outcomes such as acute therapies, rehabilitation, and cognitive impairment/dementia, which is why these factors could not be accounted for in the analyses. Moreover, we did not have genotypic data for all the 156 variants included in the previously reported PRS for mLOY, and some of the included variants were not directly genotyped, but imputed. Furthermore, although both drawn from populations of mainly European ancestry, we cannot exclude differences in ethnic background between the GISCOME and the UK biobank cohort for which the PRS was originally constructed. Finally, because the mechanisms underlying the observed associations are not known, there is a clear risk of unmeasured confounding.

To conclude, we found that common genetic variants predisposing to loss of chromosome Y in leukocytes were associated with functional outcome after ischemic stroke. This association was not restricted to the male population, rather the association tended to be stronger in women, indicating a mechanism that operates in both sexes. Based on previous literature, we speculate that the inherited genetic variants constituting the PRS affect recovery after stroke potentially through a predisposition to a genomic instability, leading to an increased risk of acquired genetic alterations, including but not limited to the sex chromosomes, which in turn could lead to immunologic impairments. Our findings need to be further evaluated in independent cohorts and mechanistically explored.

## Glossary

CH: clonal hematopoiesis
CI: confidence interval
GISCOME: Genetics of Ischemic Stroke Functional Outcome
GWAS: genome-wide association study
mCA: mosaic chromosomal alteration
mLOY: mosaic loss of chromosome Y
mRS: modified Rankin Scale
OR: odds ratio
PRS: polygenic risk score
